# Study on the Influence of Hygrothermal Aging on the Mechanical Properties of Carbon Fabric/Polyetheretherketone Composites

**DOI:** 10.3390/polym17060724

**Published:** 2025-03-10

**Authors:** Xiangyu Xu, Baoyan Zhang, Fenghui Shi, Kai Liu, Gongqiu Peng, Liang Gao, Junpeng Gao, Yu Du

**Affiliations:** 1 AVIC Manufacturing Technology Institute Composite Technology Center, Beijing 101300, China; 2 School of Aerospace Engineering, Beijing Institute of Technology, Beijing 100081, China

**Keywords:** PEEK, thermoplastic composite, hygrothermal aging, mechanical properties, CFRP, carbon fabric

## Abstract

Owing to its superior mechanical properties and recyclability, the carbon fabric/polyetheretherketone (CFF/PEEK) composite has seen increasing application in engineering domains. However, studies examining the impact of hygrothermal aging on its performance remain relatively limited in the existing literature. To investigate its durability in hygrothermal environments, this study fabricated CFF/PEEK composites with a fiber volume fraction of 55 vol% and subjected them to equilibrium hygroscopic treatment at 70 °C. The hygroscopic behavior of polyetheretherketone (PEEK) resin and CFF/PEEK composites, along with their tensile and compressive properties under dry conditions at room temperature (RTD) and wet conditions at 70 °C (ETW), were systematically evaluated. The results indicated that both PEEK resin and CFF/PEEK composites exhibited Fickian diffusion behavior during the initial stages of aging but diverged in later stages. The equilibrium moisture absorption rates were approximately 0.32% for PEEK resin and 0.19% for CFF/PEEK composites. After aging at 70 °C, the strength of both materials decreased significantly, while the modulus showed only minor changes. Under ETW conditions, the tensile strength retention rate of PEEK resin was 74.92%, and the compressive strength retention rate was 81.85%. For the CFF/PEEK composites, the tensile strength retention rate was approximately 85%, and the compressive strength retention rate was about 95%. The typical failure modes of CFF/PEEK composites did not exhibit notable differences between tensile and compressive specimens after hygrothermal aging. Resin debonding was observed in the moisture-absorbed composite specimens, while no microcracks or delamination were detected. The degradation of mechanical properties is predominantly attributed to the deterioration of the resin matrix and interface characteristics, which are caused by water molecule intrusion and the adverse effects of wet strain mismatch between the resin and fibers.

## 1. Instruction

Carbon fiber reinforced polymer composites (CFRP) have found extensive applications in the aerospace, marine, automotive, and wind power industries due to their exceptional properties such as high strength, low density, superior corrosion resistance, and fatigue endurance [1,2,3]. Since the initial commercialization of CFRP in the 1960s, its application in aircraft has progressively evolved from non-load-bearing structure components to main load-bearing structural components. The usage of CFRP in aircrafts has become a key indicator of advanced aircraft design [4,5,6]. Notably, in large commercial aircraft such as the Boeing 787 and Airbus A350, the weight fraction of CFRP exceeds 50% [7,8,9]. CFRP can be classified into the following two major categories based on their various resin matrices: thermosetting CFRP and thermoplastic CFRP. Currently, thermosetting CFRP dominate the engineering applications in aviation structure components [10,11]. However, with the continuous improvement of requirements for composite materials in the aviation field in terms of performance, low cost, and environmental protection, the drawbacks of thermosetting CFRP have become increasingly prominent. Compared with thermosetting CFRP, thermoplastic CFRP have unique advantages such as recyclability, strong designability, repairability, and easy storage, while also possessing excellent mechanical properties such as high impact resistance, damage tolerance, and high toughness [12,13]. Therefore, thermoplastic CFRP have become a new research hotspot in recent years, and they have gradually been put into practice in aerospace, automotive, marine, bioengineering, and other fields.

Currently, thermoplastic resins utilized in the aviation industry primarily consist of high-temperature resistant and high-performance resin matrices, such as polyetheretherketone (PEEK), polyphenylene sulfide (PPS), polyetherimide (PEI), polyetherketoneketone (PEKK), and polyethersulfone (PES) [14,15,16]. PEEK is a novel semi-crystalline aromatic thermoplastic engineering polymer that was successfully developed by Imperial Chemical Industries (ICI) in the late 1970s. Owing to its exceptional thermal stability, radiation resistance, corrosion resistance, superior dimensional stability, and electrical properties, as well as its outstanding processability, PEEK is widely acknowledged as one of the best-performing thermoplastic materials globally [17,18]. Currently, PEEK finds extensive application in defense and military industries, aerospace, electronics, energy development and processing, automotive manufacturing, home appliance production, and medical and healthcare sectors.

When utilized in harsh environments, the mechanical properties of resin matrix composites can be substantially diminished, thereby impacting the service life of structural components. Hygrothermal aging is a critical factor influencing the service performance of composite materials and represents one of the most significant failure modes for resin matrix composites [19,20]. In hot and humid conditions, water molecules gradually diffuse into the resin matrix and penetrate along micro-cracks to reach the fiber surface, leading to a reduction in both the mechanical properties of the resin and fibers, as well as the degradation of the interface between them, ultimately resulting in material failure. The diffusion behavior of water molecules into the interior of materials significantly affects the durability of CFRPs in engineering applications [21]. Numerous scholars have investigated the mechanical properties of CFRP in various hygrothermal environments. Ma et al. [22] reported that both the carbon fiber reinforced PEEK and PPS composites exhibit good moisture/temperature resistance and property retention after hygrothermal exposure. Wu et al. [23] found that the long-term mechanical property retention rate of CFRP was superior to that of flax fiber/epoxy resin composites and glass fiber/epoxy resin composites. Li et al. [24] reported that after the dehumidification treatment of the samples that absorbed moisture for 56 days, the retention rate of the interlaminar shear strength recuperated from 77.40% to 91.92%, and the proportion of reversible damage is 63.98%. Feng et al. [25] reported that the tensile strength of T700 carbon fiber/3228 polyurethane resin composites decreased by 14.63%, following aging at 70 °C and 85% relative humidity for one week. Wan et al. [26] found that hygrothermal aging has a significant detrimental effect on the tension–tension fatigue behavior of the 3D5D hybrid braided composites. Micelli et al. [27] noted that each type of FRP has unique components and manufacturing processes. Consequently, conclusions drawn for one material may not be directly applicable to others. However, due to the nascent stage of thermoplastic composites in engineering applications, there is limited data available on the mechanical properties of CFF/PEEK composites, and a lack of comprehensive durability test databases.

In this study, the CFF/PEEK composite and PEEK resin were subjected to saturated hygroscopic treatment at 70 °C. Subsequently, tensile tests and compressive tests were conducted on the samples both before and after the hygroscopic treatment, yielding mechanical property data under varying environmental conditions. The hygroscopic behavior of PEEK resin and CFF/PEEK composites was analyzed, and a comparative assessment was made of the mechanical properties, failure modes, and fracture morphology of the samples before and after hygroscopic exposure. This research aims to investigate the impact of hygrothermal environments on the mechanical properties of CFF/PEEK composites and provide practical guidance for the engineering applications of thermoplastic composites.

## 2. Materials and Methods

### 2.1. Raw Materials and Sample Preparation

**Figure 1:**
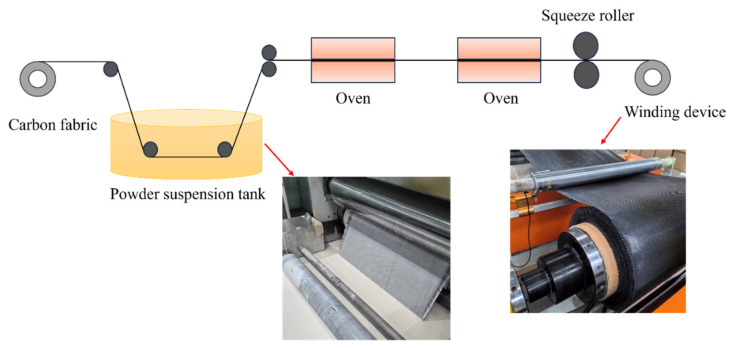
Schematic diagram of CFF/PEEK prepreg preparation process.

Satin carbon fabric and PEEK resin were utilized as raw materials to prepare CFF/PEEK thermoplastic prepreg. This thermoplastic prepreg was manufactured by the AVIC Manufacturing Technology Institute Composite Technology Center. The PEEK resin was supplied by Zhejiang Pfulon Advanced Materials Co., Ltd. (Quzhou, Zhejiang, China). The satin carbon fabric, with a unit area mass of 293 ± 8 g/m^2^, was provided by Weihai Expand Fiber Co., Ltd. (Weihai, Shandong, China). The fabric form and the finished prepreg are illustrated in Figure 1.

The mechanical specimens of PEEK resin were fabricated by injection molding and prepared in accordance with the dimensions specified by the relevant testing standards. The CFF/PEEK composite laminates were manufactured using a hot-pressing molding process. The fiber volume fraction of the composite laminates was approximately 55 vol%, and the layering process was conducted manually. The layering configurations and dimensions for various test specimens are detailed in Table 1. Following the lay-up of the prepregs, they were subjected to heating and pressing in a thermal press, resulting in the formation of composite laminates. The physical properties of CFF/PEEK composite laminates are presented in Table 2. The composite laminates were machined to meet the dimensional requirements stipulated by the testing standards. Prior to being placed in a hygroscopic environment, the edge banding of the composite samples is required.

### 2.2. Hygrothermal Treatment

In accordance with ASTM D5229, the water absorption behavior of CFF/PEEK composite specimens (50 mm × 50 mm × 2 mm) was evaluated using gravimetric analysis [32]. Initially, the specimens were subjected to drying in an air-drying oven at 40 °C, with periodic weighing conducted every 24 h. The mass loss of the laminate was calculated using Equation (1). Drying was terminated when the daily mass change stabilized below 0.02%, after which the specimens were transferred to a desiccator for further use.

(1) $$M_{i}=\frac{m_{0}-m_{i}}{m_{0}}\times 100\%$$

where *M_i_* is the dehumidification amount of the sample; and *m*_0_ and *m_i_* are the measured masses of the sample before and after drying, respectively.

The dried samples were placed in the MHU-408CRSA constant temperature and humidity chamber manufactured by Dongguan Xinbao Instrument Co., Ltd. (Dongguan, Guangdong, China). The samples underwent hygrothermal moisture absorption treatment under conditions of 85% relative humidity and 70 °C ambient temperature. At each aging interval, the samples were removed from the chamber, surface moisture was carefully blotted dry with absorbent paper, and subsequently weighed using an electronic balance with a precision of 0.1 mg. The moisture absorption amount was then calculated according to Formula (2). This procedure was repeated for five samples per group, and the mean value was recorded.

(2) $$W_{i}=\frac{G_{i}-G_{0}}{G_{0}}\times 100\%$$

where *W_i_* is the moisture absorption capacity of the sample; *G_i_* is the mass of the sample after moisture absorption; and *G*_0_ is the mass of the sample after drying.

### 2.3. Mechanics Performance Testing

The tensile properties of PEEK resin were evaluated in accordance with the GB/T 1040-2018 standard, while the compressive properties were assessed following the GB/T 1041-2008 standard [33,34]. For CFF/PEEK composites, the tensile properties were tested according to ASTM D3039, and the compressive properties were determined based on ASTM D6641 [35,36]. Prior to the commencement of the mechanical test, strain gauges should be bonded to the working section of the specimens to monitor its strain. All tests were conducted using an Instron-5982 universal testing machine (Instron Instruments Ltd., Boston, MA, USA). The tensile specimens were loaded at a rate of 3 mm/min, and the compressive specimens were subjected to a loading rate of 1 mm/min. The test environment temperature was controlled using a WGDN-7350 high- and low-temperature chamber produced by Lishi (Shanghai) Scientific Instrument Co., Ltd. (Shanghai, China). To ensure data reliability, the mechanical properties of three batches of composites were evaluated, with each batch comprising no fewer than six valid samples. The test results were normalized based on a nominal thickness of 2.4 mm.

### 2.4. Morphological Analysis

The microscopic morphologies of the fracture surfaces of the mechanical specimens were examined both before and after hygrothermal aging using a Quanta 450 FEG field emission environmental scanning electron microscope (SEM) produced by Thermo Fisher Scientific Ltd. (Waltham, MA, USA). Prior to SEM observation, it was crucial to improve the conductivity of the specimens to ensure optimal imaging quality. This was accomplished by applying a gold coating via sputtering for 60 s using a gold sputtering device.

## 3. Results and Discussion

### 3.1. Moisture Absorption Characteristics

The pure PEEK resin and the CFF/PEEK composite laminate specimens were subjected to saturated moisture absorption treatment at 70 °C, respectively. The diffusion behavior of water molecules within the composite material can be characterized by the water transport kinetics equation as follows:

*M_t_*/*M_m_* = *kt^n^* (3)

where *M_t_* represents the moisture absorption rate at time *t*, *M_m_* denotes the saturated moisture absorption rate, *k* signifies the diffusion constant of water molecules, and *n* is a critical parameter that characterizes the swelling mechanism of the material.

The *k* and *n* obtained from fitting the moisture absorption data of PEEK resin and the composite materials using the logarithmic form of the aforementioned equation are summarized in Table 3. Analysis of these data indicates that the values of *n* for both moisture absorption curves are approximately 0.5. Therefore, it can be reasonably inferred that the initial water absorption behavior of the PEEK resin and CFF/PEEK composite during the early stages of aging is consistent with the Fickian diffusion model [37].

The solution of the Fickian equation can be described by Equation (4).

(4) $$\frac{M_{t}}{M_{m}}=1-\frac{8}{\pi^{2}}\sum_{n=0}^{n=\infty }\frac{1}{{\left(2n+1\right)}^{2}}exp\left[-\left(\frac{Dt}{h^{2}}\right)\pi^{2}{\left(2n+1\right)}^{2}\right]$$

where *D* is the diffusion coefficient of the material and *h* is the thickness of the specimen.

When *M_t_*/*M_m_* ≤ 0.6, the initial part of the water absorption curve can be described by Equation (5).

(5) $$\frac{M_{t}}{M_{m}}=\frac{4}{h}\sqrt{\frac{Dt}{\pi }}$$

When *M_t_*/*M_m_* > 0.6, the water absorption curve can be described by Equation (6).

(6) $$\frac{M_{t}}{M_{m}}=1-exp\left[-7.3{\left(\frac{Dt}{h^{2}}\right)}^{0.75}\right]$$

The diffusion coefficient *D* of water molecules in the material can be given by Equation (7).

(7) $$D=\frac{\pi }{{\left(4Mm\right)}^{2}}{\left(\frac{M_{t}h}{\sqrt{t}}\right)}^{2}=\pi {\left(\frac{hk^{\prime }}{4M_{m}}\right)}^{2}$$

where *k′* represents the slope of the linear part of the equation.

**Figure 2:**
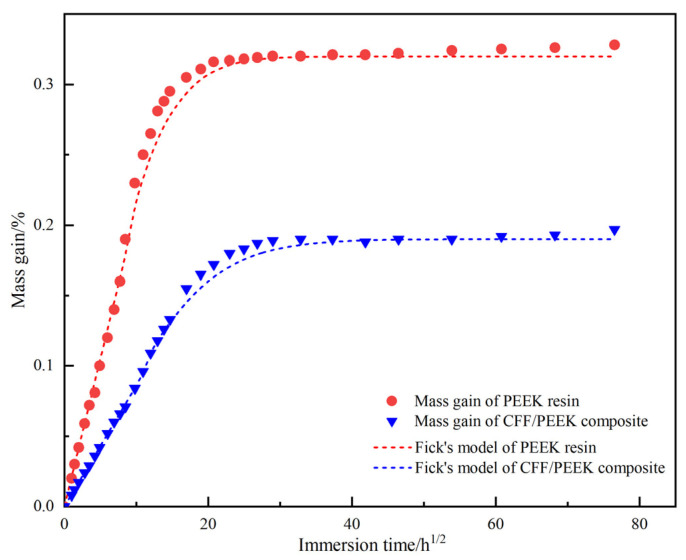
Water absorption curves and Fickian fitting results of PEEK resin and CFF/PEEK composite.

Based on the parameters derived from Table 2 of the experiment, the Fickian model fitting the curves for the PEEK resin and CFF/PEEK composite materials were generated and compared with the measured moisture absorption data, as illustrated in Figure 2. The distribution of the fitting curves and data points in Figure 2 indicates that during the initial stage of moisture absorption, the curve gradient is relatively steep, indicating a linear relationship between the moisture absorption rate and *t^1/2^*. The experimental data exhibit excellent agreement with the Fickian model. In the later stages of moisture absorption, the curve deviates from linearity. This deviation can primarily be attributed to the combined effects of temperature and humidity, which cause both the resin itself and internal defects within the composite material to absorb water, leading to the rapid diffusion of water molecules within the composite material. Following this rapid absorption phase, the moisture absorption rate gradually decreases, and the moisture absorption curve flattens, eventually reaching saturation. The equilibrium moisture absorption rates for the PEEK resin and CFF/PEEK composite material are approximately 0.32% and 0.19%, respectively. Given that carbon fibers mainly consist of carbon elements with a disordered graphite structure [38], they exhibit minimal water absorption. Consequently, in the composite material, only the matrix and interface regions absorb water, resulting in significantly lower saturation moisture absorption for the CFF/PEEK compared to the pure PEEK resin.

### 3.2. Influence of the Hygrothermal Environment on the Tensile Properties

**Figure 3:**
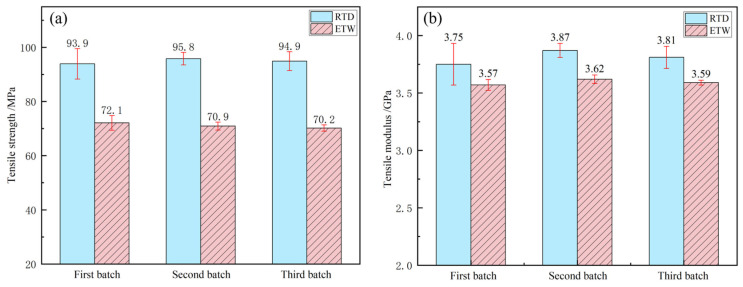
Tensile properties of PEEK resin: (**a**) tensile strength; (**b**) tensile modulus.

**Figure 4:**
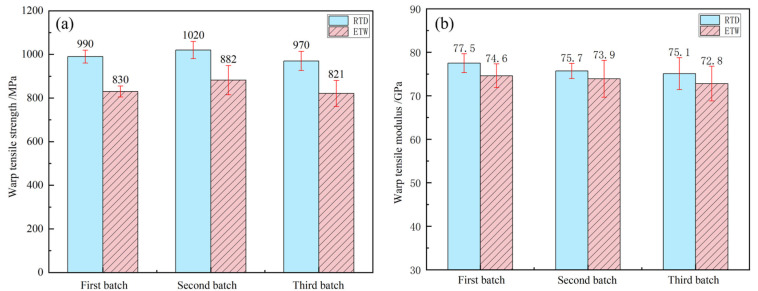
Warp tensile properties of CF/PEEK composite: (**a**) tensile strength; (**b**) tensile modulus.

**Figure 5:**
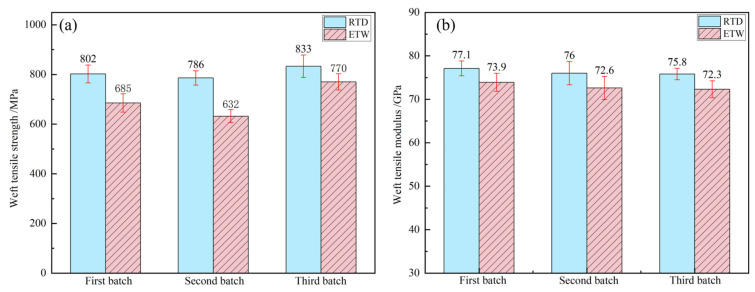
Weft tensile properties of CF/PEEK composite: (**a**) tensile strength; (**b**) tensile modulus.

The tensile properties of PEEK resin under dry conditions at room temperature (RTD) and wet conditions at 70 °C (ETW) are illustrated in Figure 3. The tensile properties of CFF/PEEK composites under RTD and ETW conditions are presented in Figure 4 (warp direction) and Figure 5 (weft direction). Analysis of the data reveals a notable reduction in tensile properties for both PEEK resin and CFF/PEEK composites under ETW conditions. Table 4 provides the retention rates of the tensile properties of PEEK resin and CFF/PEEK composites. Specifically, the tensile strength of PEEK resin under ETW conditions is approximately 74.92% of that observed under RTD conditions. For CFF/PEEK composites, the warp tensile strength is approximately 84.98%, and the weft tensile strength is approximately 86.08% of their respective RTD values. In contrast, the tensile modulus exhibits less sensitivity to hygrothermal effects. Under ETW conditions, the tensile modulus of PEEK resin is approximately 94.32% of its RTD value, while for CFF/PEEK composites, the warp tensile modulus is approximately 96.94%, and the weft tensile modulus is approximately 95.59% of their RTD values.

The significant influence of the hygrothermal environment on the tensile strength of the composites can be attributed to multiple factors. Firstly, in a wet state, water molecules that penetrate the resin matrix can degrade both the performance of the resin and the interface between the fibers and matrix. Furthermore, water molecules infiltrating through micro-cracks in the resin and reaching the carbon fiber surface can reduce the tensile strength of the fibers to some extent [39]. Additionally, elevated temperatures exacerbate this issue by accelerating water molecule penetration into the composite materials and directly damaging the molecular chains of the resin matrix, thereby weakening the bonding between the matrix and fibers, and reducing the tensile strength of the composites. Moreover, the combined effects of temperature increase and moisture intrusion can cause differential expansion between the fibers and matrix due to differences in their thermal and wet expansion coefficients, leading to internal stresses within the composites, which may contribute significantly to the reduction in tensile strength under ETW conditions. In contrast, hygrothermal environments impact the tensile modulus of composites to a lesser extent. This is because the elastic modulus of carbon fibers remains largely unaffected by temperature and humidity within a certain range, while the modulus of the PEEK resin matrix decreases at high temperatures and under wet conditions. Consequently, the tensile modulus of composites, which is primarily determined by the elastic modulus of carbon fibers, is generally less influenced by hygrothermal environments.

**Figure 6:**
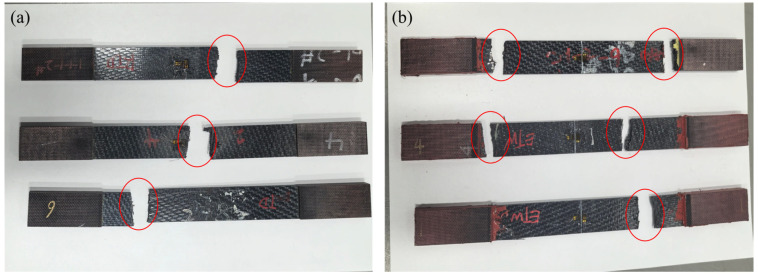
Typical failure modes of tensile test for CFF/PEEK composite: (**a**) CTD; (**b**) ETW.

**Figure 7:**
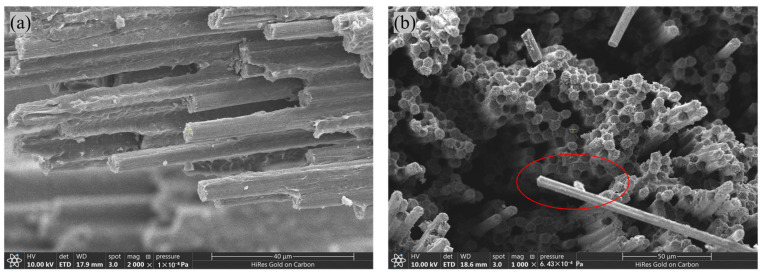
Morphologies of CFF/PEEK composite tensile fracture under different environmental conditions: (**a**) RTD; (**b**) ETW.

The failure modes of tensile specimens of CFF/PEEK composites are illustrated in Figure 6, while the microscopic morphology of the fracture surfaces is depicted in Figure 7. As observed from Figure 6, tensile failures occur predominantly in the central or terminal regions of the working section, indicating a consistent and effective failure mode. The fracture surfaces exhibit a relatively uniform appearance, with no significant differences noted between the ETW and RTD states. Microscopic examination of the fracture surfaces in Figure 7 reveals that, after moisture absorption, the failure mechanisms involve fiber pull-out and fracture, maintaining a neat fracture surface. Additionally, no evident delamination and cracks were observed after moisture absorption, suggesting minimal chemical alterations during hygrothermal aging. In summary, the hygrothermal aging mechanism, affecting the tensile properties of CFF/PEEK composites following 70 °C saturated moisture absorption treatment, primarily involves the degradation of the matrix and interface due to moisture ingress, as well as property deterioration caused by the differential wet strain between the resin and fibers, leading to wet stress [40].

### 3.3. Influence of Hygrothermal Environment on Compression Properties

**Figure 8:**
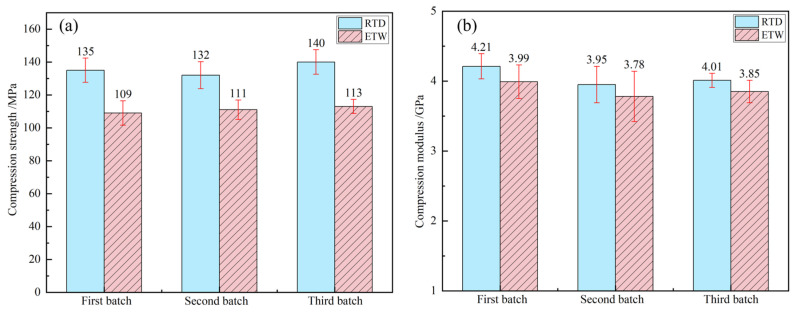
Compression properties of PEEK resin: (**a**) compression strength; (**b**) compression modulus.

**Figure 9:**
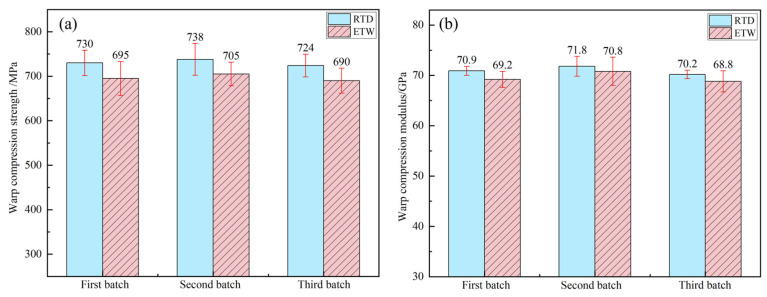
Warp compression properties of CF/PEEK composite: (**a**) compression strength; (**b**) compression modulus.

**Figure 10:**
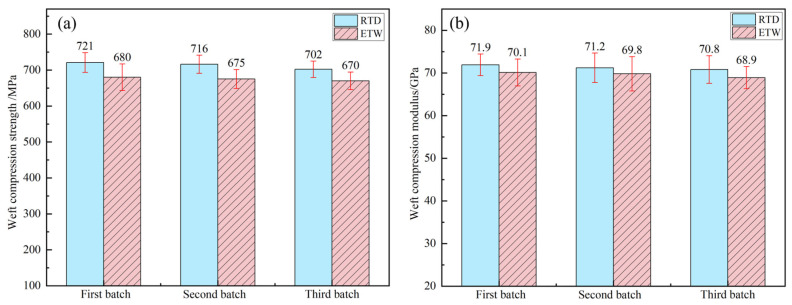
Weft compression properties of CF/PEEK composite: (**a**) compression strength; (**b**) compression modulus.

The compression properties of PEEK resin under RTD conditions and ETW conditions are illustrated in Figure 8. The compressive properties of CFF/PEEK composites under these same conditions are depicted in Figure 9 (warp direction) and Figure 10 (weft direction). Table 5 presents the compression property retention rates for both PEEK resin and CFF/PEEK composites. Compared to tensile strength, the compression strength of PEEK resin and CFF/PEEK composites exhibits less sensitivity to hygrothermal environments. Meanwhile, the changes in the compression modulus mirror those of the tensile modulus, with both demonstrating relatively high retention rates following hygrothermal aging. Under ETW conditions, the compression strength of PEEK resin is approximately 81.85% of its RTD value, while the compressive modulus retains about 95.49% of its RTD value. For CFF/PEEK composites under ETW conditions, the compressive strength in the warp direction is approximately 95.35% of its RTD value, and in the weft direction, it is about 94.67% of its RTD value. Similarly, the compressive modulus in the warp direction is approximately 98.07% of its RTD value, and in the weft direction, it is about 97.62% of its RTD value.

Under ETW conditions, the compressive performance of PEEK resin exhibits a significant reduction, whereas the compressive strength of CFF/PEEK composite laminates remains relatively stable. This phenomenon can be attributed to water molecules infiltrating the resin matrix in a wet environment, which leads to a marked decline in the compressive properties of the resin matrix and consequently affects the overall compression performance of the composite laminates. Moreover, water molecules also penetrate the interlaminar and carbon fiber/resin interfaces within the composites, causing interface degradation and diminishing their load transfer capability, thereby further compromising their compressive performance [41,42]. The compression strength of composites under hygrothermal conditions serves as a direct indicator of the impact of hygrothermal aging on the compressive strength of the resin matrix.

**Figure 11:**
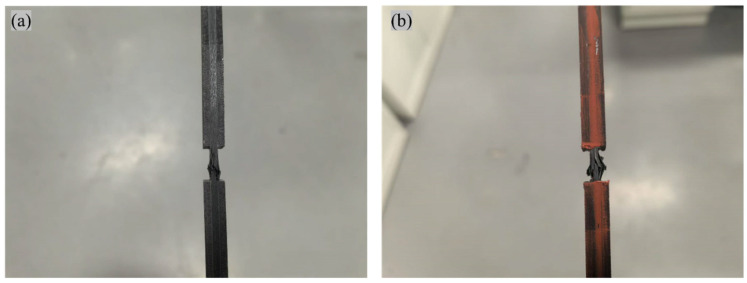
Typical failure modes of compression test for CFF/PEEK composite: (**a**) RTD; (**b**) ETW.

**Figure 12:**
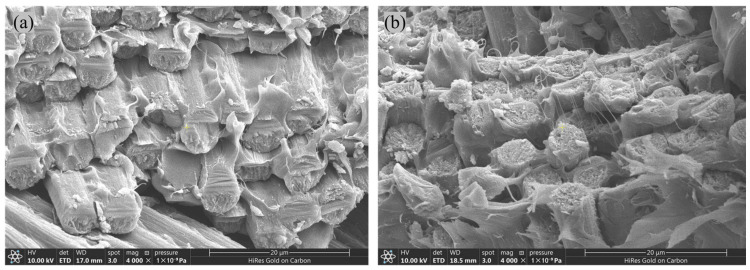
Morphologies of CFF/PEEK composite compression fracture under different environmental conditions: (**a**) RTD; (**b**) ETW.

The failure modes of the compression specimens of CFF/PEEK composites are illustrated in Figure 11, while the fracture surface morphologies are depicted in Figure 12. From Figure 11, it is evident that the compression specimens failed within the working section, exhibiting an effective failure mode. Minor delamination was observed at the failure location, and the fracture surfaces displayed wedge-shaped characteristics. No significant differences were noted in the failure modes between the RTD and ETW states. As shown in Figure 12, which presents the fracture surface damage morphology of the specimens, the fracture surfaces before and after moisture absorption remained relatively flat, with no apparent microcracks or delamination. Consequently, it can be inferred that following the 70 °C hygrothermal aging process, no significant chemical reactions occurred in the CFF/PEEK composites. The reduction in compression performance is primarily attributed to the influence of water molecules on the resin matrix properties and the degradation of interfacial performance.

## 4. Conclusions

This study investigates the impact of the hygrothermal environment on the mechanical properties of CFF/PEEK composites, examining the changes in these properties before and after moisture absorption. The saturated moisture absorption rate of CFF/PEEK composites at 70 °C is approximately 0.19%, compared to approximately 0.32% for pure PEEK resin. The initial moisture absorption behavior of both materials adheres to Fick’s law, although deviations occur in the later stages. After absorbing moisture, the tensile strength retention rate of PEEK resin is approximately 74.92%, while that of CFF/PEEK composites is approximately 84.98% in the warp direction and 86.08% in the weft direction. In contrast, the tensile modulus exhibits less sensitivity to the hygrothermal environment. The compression strength retention rate of PEEK resin is approximately 81.85%, while the compression modulus retention rate stands at approximately 95.49%. For CFF/PEEK composites, compressive performance is relatively less affected by the hygrothermal environment. It was found that the damage mode of CFF/PEEK composites under the ETW state is not significantly different compared to that of the RTD state. Resin debonding was observed in the moisture-absorbed composite specimens, while no microcracks or delamination were detected. The primary reasons for the degradation of the mechanical properties of the CFF/PEEK composites after moisture absorption at 70 °C are attributed to the deterioration of the resin matrix and interface performance, as well as the wet stress induced by the inconsistent wet strain between the resin and fibers.

## Figures and Tables

**Table 1:** Test specimen layup of CFF/PEEK composite laminates.

Type	Ply Count	Layup	Nominal Lamina Thickness/mm	Size/mm
Warp Tensile Test	8	[0_f_]_8_	2.4	250 × 25
Weft Tensile Test	8	[90_f_]_8_	2.4	250 × 25
Warp Compression Test	8	[0_f_]_8_	2.4	140 × 12
Weft Compression Test	8	[90_f_]_8_	2.4	140 × 12

Notes: Subscript f—fabric prepreg; layup 0 or 90—angle between the fabric warp direction and the *x*-axis direction.

**Table 2:** Physical properties of CFF/PEEK composite laminates.

Properties	Resin Content	Density	Fiber Volume Fraction	Glass Transition Temperature
Value	38 wt%	1.57 g/cm^3^	55 vol%	146 °C
Test Method	GB/T 3855 [28]	GB/T 1463 [29]	ASTM D3171 [30]	ASTM D 7028 [31]

**Table 3:** Summary of water absorption parameters.

Material	*n*	*k*/h^−*n*^	*M_m_*/%	*k′*	*D/*(10^−3^ mm^2^/h^−1^)
PEEK	0.455	0.057	0.32	0.008	2.317
CFF/PEEK composite	0.526	0.063	0.18	0.021	1.353

Notes: *n*—parameter describing the swelling mechanism; *k*—diffusion constant; *h*—specimen thickness, *M_m_*—maximum moisture uptake at equilibrium state; $k^{\prime }$—slope of linear portion of the sorption curves; D—diffusion coefficient of the composites.

**Table 4:** Retention rate of the tensile properties of PEEK resin and CFF/PEEK composites.

Material	Performance	First Batch	Second Batch	Third Batch	Average
PEEK resin	Tensile strength (%)	76.78	74.01	73.97	74.92
	Tensile modulus (%)	95.20	93.54	94.23	94.32
CFF/PEEK composite	Warp tensile strength (%)	83.84	86.47	84.64	84.98
	Warp tensile modulus (%)	96.26	97.62	96.94	96.94
	Weft tensile strength (%)	85.41	80.41	92.44	86.08
	Weft tensile modulus (%)	95.85	95.53	95.38	95.59

**Table 5:** Retention rate of compression properties of PEEK resin and CFF/PEEK composites.

Material	Performance	First Batch	Second Batch	Third Batch	Average
PEEK resin	Compression strength (%)	80.74	84.09	80.71	81.85
	Compression modulus (%)	94.77	95.70	96.01	95.49
CFF/PEEK composite	Warp compression strength (%)	95.21	95.53	95.30	95.35
	Warp compression modulus (%)	97.60	98.61	98.01	98.07
	Weft compression strength (%)	94.31	94.27	95.44	94.67
	Weft compression modulus (%)	97.50	98.03	97.32	97.62

## Data Availability

All supporting data are contained within the manuscript.
